# Effect of Glucocorticoids on Ultrastructure of Myocardial Muscle in the Course of Experimentally Induced Acute Myocardial Ischemia

**DOI:** 10.1155/2017/2108497

**Published:** 2017-07-16

**Authors:** Piotr Kuropka, Maciej Dobrzyński, Andrzej Gamian, Kinga Gostomska-Pampuch, Jan Kuryszko, Ireneusz Całkosiński

**Affiliations:** ^1^Department of Histology and Embriology, Wroclaw University of Environmental and Life Sciences, Norwida 31, 50-375 Wrocław, Poland; ^2^Department of Conservative Dentistry and Pedodontics, Wroclaw Medical University, Krakowska 26, 50-425 Wrocław, Poland; ^3^Department of Medical Biochemistry, Wroclaw Medical University, Chałubińskiego 10, 50-368 Wrocław, Poland; ^4^Institute of Immunology and Experimental Therapy, Polish Academy of Sciences, Weigla 12, 53-114 Wroclaw, Poland; ^5^Laboratory of Neurotoxicology and Environmental Diagnostics, Wroclaw Medical University, Bartla 5, 51-618 Wroclaw, Poland

## Abstract

The search for effective methods of myocardial cytoprotection against ischemia is the most significant issue in modern cardiology and cardiac surgery. Glucocorticoids are deemed very strong modulators of inflammatory response and thus can potentially protect heart muscle from postreperfusion injury and myocardial ischemia during cardiac surgery. Ultrastructural examination of the left ventricle heart samples revealed that the intravenous application of dexamethasone and hydrocortisone proved to exert cytoprotective effect on cardiomyocytes during experimentally induced acute ischemia in rats.

## 1. Introduction

The search for effective methods of myocardial cytoprotection against acute and chronic ischemia is the most desirable and relevant issue in modern cardiology and especially cardiac surgery. The protection of myocardial muscle from ischemia-induced left ventricular dysfunction during coronary artery bypass grafting, valve surgery, or heart transplantation is an essential matter for further success of the cardiac procedure. With age, this protection is reduced by altered mitochondrial metabolism. The defective mitochondria persist in the aged heart, leading to enhanced oxidant production and oxidative injury and the activation of oxidant signaling for cell death [1].

The extracorporeal circulation (on-pump operations, ECC) and surgical trauma (chest cutting) together with vigorous release of bacterial lipopolysaccharide (LPS) during cardiac surgery are very strong activators of immunological and inflammatory response (systemic inflammatory response syndrome, SIRS) [2, 3]. The activated monocytes produce a huge quantity of proinflammatory interleukins (IL-1, Il-2, Il-6) and Tumor Necrosis Factor alpha (TNF*α*). The aforementioned cytokines enhance production of adhesion molecules (VCAM, ICAM, selectin E, P, and L) and their presentation on the surface of endothelial cells. The adhesion molecules allow lymphocytes to adhere to the vessel wall and then migrate from the blood into the vascular wall. Within the vessel wall lymphocytes become disintegrated, which strengthens permeability of the vasculature and causes vascular leakage. Concomitantly activated complement enhances this pathological process. All these factors and processes trigger the multiorgan damage, including lungs, liver, brain, and kidneys [3]. Hence, SIRS can be also a source of detrimental dysfunction of heart and poor prognosis for the patients after seemingly successful operation. In this context the role of glucocorticoids as a powerful modulator of immunological and inflammatory response in the cardiac protection during ECC has been postulated [4, 5]. This is because activation of glucocorticoid receptor can prevent cardiac injury through transcriptional activation of Bcl-xL gene which inhibits apoptosis in cardiomyocytes [6]. The study was supposed to answer the question whether intravenously administered glucocorticoids (dexamethasone, hydrocortisone) during acute myocardial ischemia are able to exert their protective effect on cardiomyocytes in rats.

## 2. Material and Methods

The study was approved by a local Ethics Committee for Animal Experiments. The study was performed on 30 Wistar male (15) and female (15) rats of body weight of 250–280 g, treated in compliance with the Guide for Care and Use of Laboratory Animals of the National Institutes of Health (NIH publication number 85-23, revised in 1985). The rats were divided into four groups: Group I: rats with induced acute myocardial ischemia; Group II: rats which were administered hydrocortisone hemisuccinate (20 mg/kg; i.v.) 15 min before the acute myocardial ischemia was induced; Group III: rats which were administered dexamethasone (0.1 mg/kg; i.v.) 1.0 h before acute ischemia; Group IV: the control group, not submitted to acute myocardial ischemia.

The acute myocardial ischemia was induced in following procedure. All the studied animals were anesthetized with thiopental in a dose of 30 mg/kg, intubated, and connected to a mechanical respirator (Surgivet, Dublin, USA). The heart action was monitored by ECG Aspekt 700 (Aspel, Zabierzów, Poland) to the moment heart arrest. The chest was cut open in the midline. Acute myocardial ischemia was induced by disconnecting the respirator and compressing the tracheal tube, which was shortly followed by heart arrest. Samples from the left ventricle were taken for electron microscopy after 15 min of heart arrest. The tissue was fixed overnight at 4°C in 2.0% glutaraldehyde and postfixed in 2.0% buffered osmium tetraoxide. After dehydration in acetone series cubic samples 0.25 mm^3^ were embedded in Epon 812 resin and ultrathin sections were stained with uranyl acetate and lead citrate and examined under a Tesla BS500 electron microscope (Tesla, Brno, Czech Republic).

To determine and compare changes in size of the mitochondria, the EM images were analyzed with Photoshop CS3, using area analysis function. For area measurement, the mitochondria were circled by the lasso tool and then the areas of the circles were calculated and converted to their actual values using the scale bar.

## 3. Results

**Figure 1:**
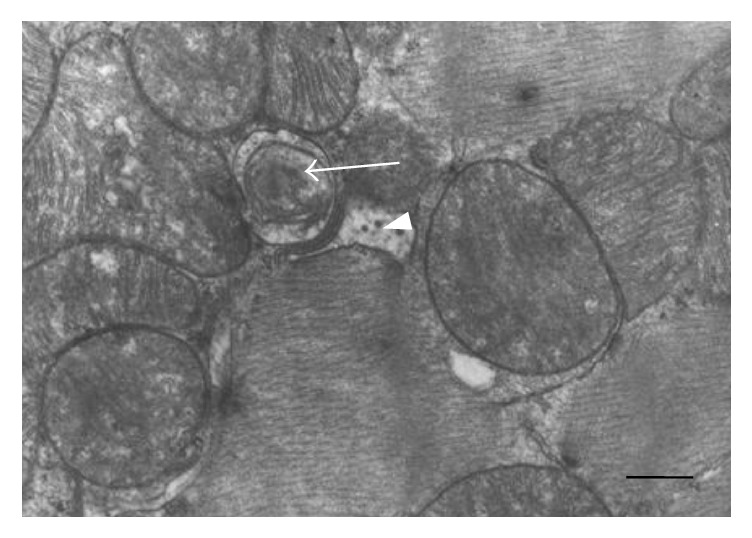
Cardiomyocyte during induced acute ischemia (group I). The numerous myelin-like structures in mitochondria (white arrow). The numerous vacuoles and multivesicular bodies in cytoplasm (white arrowhead). The mitochondrial matrix is dark and mitochondrial crests are not clearly visible. Magnification 6000x. Scale bar: 500 nm.

The vast pathological changes in the ultrastructure of cardiomyocytes were observed in group I. The previously well-marked areas of euchromatin and heterochromatin within the structure of cellular nuclei became diffused. The following changes were also found during the electron microscope evaluation: clearly visible enlargement of the area of heterochromatin in the periphery of nuclei, atrophy and shortening of mitochondrial crests, mitochondrial inclusions, and disruption of mitochondrial structure. Beyond that the enlargement of Golgi cisterns and endoplasmatic reticulum, distortion, and further disintegration of myofilament structure were observed (Figure 1). The cytoplasm of cardiomyocytes was also characterized by presence of distinct myelin structures, fat deposits, lipofuscin granules, and numerous secondary lysosomes (cytolysis phenomenon).

However, the glycogen granules were not visualized within the cytoplasm of cardiomyocytes.

In the groups II and III the main changes were associated with sarcolemma and mitochondria. Myelin-like structures and some vacuoles in cytoplasm, giant mitochondria formation, and enlargement of intercellular space in intercalated disk were observed.

**Figure 2:**
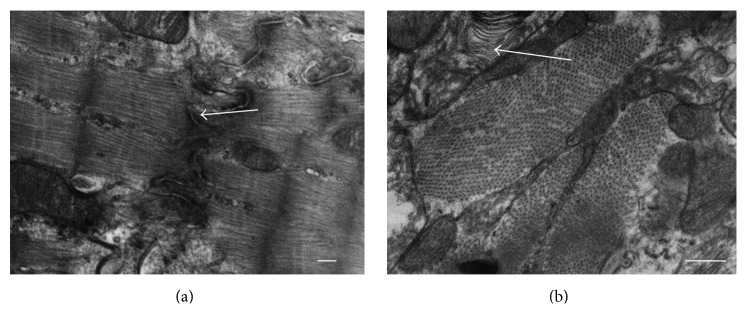
Cardiomyocyte during induced acute ischemia (group II). (a) The enlarged intercellular space within intercalated discs (arrow). The myelin-like structures in sarcoplasm. Magnification 10000x. Scale bar: 500 nm. (b) Fusion of mitochondria, formation of giant mitochondria, and myelin-like structures within cardiomyocyte cytoplasm (arrow). Magnification 6000x. Scale bar: 500 nm.

**Figure 3:**
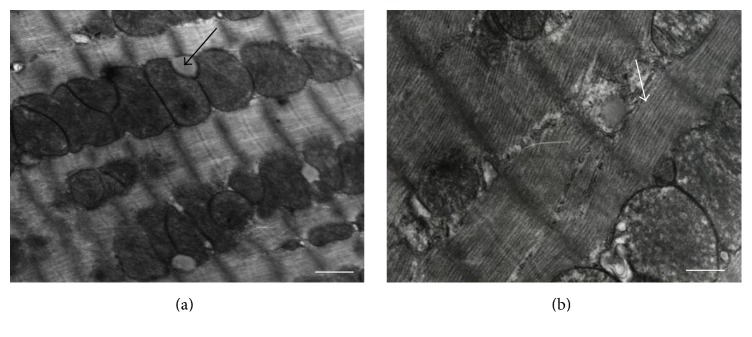
Cardiomyocyte during acute ischemia (group III). (a) Mitochondria have easily visible crests. Few myelin structures and vacuoles in sarcoplasm (arrow). Magnification 6000x. Scale bar: 500 nm. (b) The irregular shape of myofilaments (arrow), but with well preserved structure of sarcomeres in the myocytes. Magnification 6000x. Scale bar: 500 nm.

**Figure 4:**
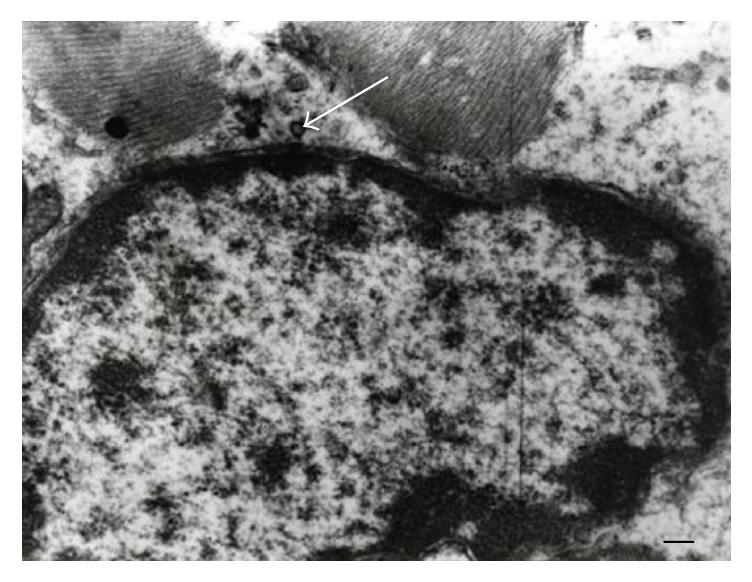
Cardiomyocyte of the control group (group IV). The numerous glycogen granules in sarcoplasm (white arrow). The myelin structures are absent. Magnification 10000x. Scale bar: 500 nm.

The morphological abnormalities found in the myocardial ultrastructure in the groups II and III, that is, aggregation and further fusion of mitochondria, were mostly reversible and not so conspicuous as compared with the group I (Figures 2 and 3). The ultrastructure of myocardium in these two last mentioned groups was quite similar to the one in the control group (group IV) (Figure 4).

In summary of the obtained results, we must point out that the devastating pathological changes in myocardial structure, especially in mitochondria, vacuolization of cytoplasm, enlargement of cell-organelle lumen (endoplasmatic reticulum, Golgi complex), enlargement of intercellular spaces within intercalated discs, and lack of glycogen granules in cytoplasm were found in rats submitted to the acute myocardial ischemia.

**Figure 5:**
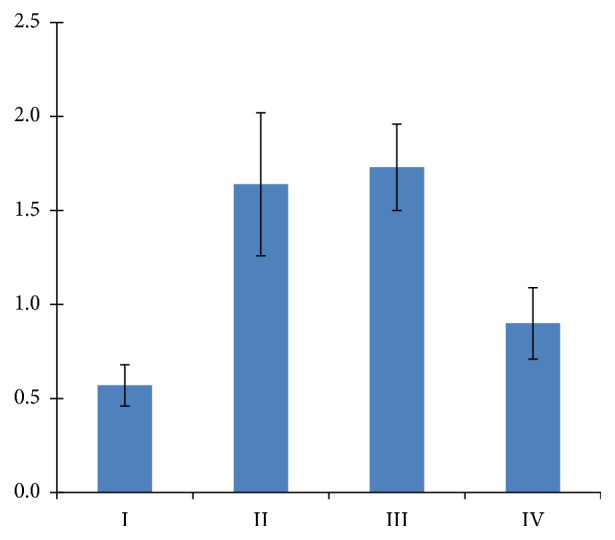
Individual area of the mitochondria in cardiomyocytes (*μ*m^2^).

Morphometric analysis revealed reduction in mitochondria size in group I whereas in groups II and III their enlargement when compared to the control group IV (Figure 5). The decrease in size was followed by increase in number of mitochondria in group I.

## 4. Discussion

The observed abnormalities in the ultrastructure of cardiomyocytes are the consequence of acute myocardial ischemia and further the enhanced anaerobic metabolism within the myocardial cells followed by significant increase in lactic acid level and intracellular acidity. During acute myocardial ischemia, the functionally impaired mitochondria do not produce sufficient quantity of ATP. The ATP deficiency disturbs osmotic balance through blocking sodium-potassium ion pump [1, 7]. All the aforementioned factors cause enlargement of cellular compartments, lysosomal membrane damage, release of proteolytic enzymes into cytoplasm, and finally cellular autolysis. The signals from mitochondria may induce cell death by apoptosis. Therefore, glucocorticoids inhibit changes in cardiomyocytes by overexpressing Bcl-xL gene thus preventing the apoptosis [1, 6].

The majority of ultrastructural changes in rats from the group I were irreversible and brought to the total destruction of myocardial cells. The cytoprotective effect of hydrocortisone and dexamethasone administered in rats from the group II and III was validated. The steroids proved to stabilize cytoplasmatic membranes and intracellular structures thus preventing formation of destructive necrotic foci in myocardial ultrastructure. Most of the pathological changes found in the myocardial ultrastructure of these two groups (group II and III) were reversible. Glucocorticoids have been well-known and generally used group of medicaments in acute and chronic inflammation-related diseases for many years. They are also supposed to be effective in treatment of postcardiac surgery SIRS [5]. In this respect, dexamethasone is deemed a very powerful modulator of inflammatory response and thus postreperfusion heart injury [8]. However, there are some controversies about the negative influence of adrenal steroids on postinfarct scar formation, postoperative respiratory dysfunction, and heart arrhythmias after cardiac surgery [9, 10]. On the other hand, steroid administration before pediatric cardiac surgery using cardiopulmonary bypass has been shown to modulate the inflammatory response and reduce myocardial injury [11]. Despite that Checchia et al. have doubts on what type, dosing, method of administration, and timing of glucocorticoids are most advisable [11]. According to our observations the cytoprotective effect of glucocorticoids was confirmed irrespective of method of the steroid administration (hydrocortisone or dexamethasone) and timing before experimentally induced myocardial ischemia. The administration of glucocorticoids both 15 minutes and 1 h before the onset of acute ischemia exerts the same cardioprotective effect as confirmed by our experiment. However, it can be assumed that the deleterious impact of acute ischemia on heart ultrastructure in the groups II and III was not found owing to relatively high dose of glucocorticoids we used in the study. On the other hand, the applied dosage protocol was much less intensive as compared with previous investigations (dosage of dexamethasone in the cited studies: 2.0 mg/kg and 6.0 mg/kg versus 0.1 mg/kg in our experiment), which also corroborated the cytoprotective activity of steroid hormones [8, 12]. Hence, we can suppose that the cardioprotective effect of glucocorticoids can be also achieved at lower quantity of administered steroid hormones thus reducing the risk of their adverse and detrimental effects on human body. Probably, the timing of glucocorticoids administration is also fundamental for the appearance of their “full-fledged” activity [11]. It is possible that a proper dose of dexamethasone, different timing of the dose, or repeated administration could have influenced the clinical course. Perhaps if a longer time had been allowed for the glucocorticoid to work, the inhibitory effect on proinflammatory cytokine production would have been greater [13]. In majority of previous clinical investigations, which showed the strong inhibition of inflammatory response by steroids during cardiac surgery and thus attenuation of postreperfusion myocardial injury, dexamethasone was administered before the onset of the procedure [13–15]. Checchia et al. [16] proved that dexamethasone administration before cardiopulmonary bypass in children resulted in a significant decrease in cardiac troponin I levels at 24 hrs postoperatively. Furthermore, the authors of the study postulate that this may represent a decrease in myocardial injury and a possible cardioprotective effect produced by dexamethasone. Referring to the literature reports and previous experiments, we could expect that the exclusive administration of dexamethasone would have a protective impact on myocardial cells [5, 8, 12]. Surprisingly, the view of myocyte ultrastructure in the group II (intravenous injection of hydrocortisone) did remain similar to the one in the group III (dexamethasone administration). Dexamethasone may also significantly reduce the inflammatory response during cardiac surgery procedure in human [15]. It is strongly hypothesized that the gradual increase in anti-inflammatory interleukin-10 level in blood observed during steroids administration is at least partially responsible for the attenuation of inflammatory reaction [15]. The inhibition of cytochrome-c release is also involved in the dexamethasone-induced cardiac protection [8]; however lysosomal disruption is an important consequence of myocardial ischemia and early treatment with dexamethasone prevents the loss of myocardial lysosomal and cellular enzymes. In this way, dexamethasone may act to retard the spread of the developing infarct within the ischemic myocardium mainly by retardation of apoptotic signals from mitochondria [6, 12]. The slight morphological changes observed in the groups II and III as compared with very severe damage of myocardial ultrastructure in the group I support the aforementioned hypothesis. As mentioned before, the vigorous release of bacterial lipopolysaccharide (LPS) during extracorporeal circulation may also contribute to myocardial cell damage found after cardiac surgery and stroke [17]. Hence, it is also postulated that the dexamethasone administration-mediated abrogation of bacterial infestation-induced cardiac dysfunction may be due to suppression of nitric oxide production [18]. In spite of undoubtedly beneficial cytoprotective impact of exogenous steroid hormones on cardiomyocytes during acute myocardial ischemia some of the adverse effects of steroid therapy must be also considered. The potential negative influence of steroids on circulatory system is related to the phenomenon of enhancement of catecholamines synthesis and simultaneous blockage of their uptake by tissues. The detrimental impact may be manifested by significant increase in the blood level of catecholamines and its proarrhythmogenic and catabolic effect on heart during ischemic stress [10, 19]. This could explain hypothetically the need to use steroid drugs concomitantly with *β*-blockers.

The results of the study demonstrated potential cytoprotective effect of steroid administration at the respectably high dosage and regardless of the timing of the dose before experimentally induced acute myocardial ischemia [20].

We need to emphasize that glucocorticoids can exert both positive and negative effects on the heart. Glucocorticoid administration improves contractile performance of the heart and inhibits cardiomyocyte apoptosis triggered by ischemia, cytokines, and cardiotoxic drugs. Conversely, elevated levels of glucocorticoids have also been linked to a variety of negative cardiac outcomes, for example, reduced heart rate in healthy human and induction of cardiac hypertrophy [21].

Some authors suggest sex differences associated with presence of testosterone and estrogen influence calcium levels in heart muscle cells [22]. We did not observe any sex differences between analyzed groups.
